# Editorial: Cardiovascular responses to exercise: clinical and pathological perspectives in athletes

**DOI:** 10.3389/fcvm.2026.1792420

**Published:** 2026-02-13

**Authors:** Timothy D. Bryson, Xiaosong Gu

**Affiliations:** 1 Department of Internal Medicine, Hypertension & Vascular Research Division, Henry Ford Health, Detroit, MI, United States; 2 Department of Physiology, Wayne State University School of Medicine, Detroit, MI, United States; 3 Department of Cardiology, The Second Affiliated Hospital of Soochow University, Suzhou, Jiangsu, China

**Keywords:** cardiac rehabilitation, cardiac remodeling, cardiovascular disease (CVD), exercise, personalized precision medicine

## Introduction

Although it is well known and accepted that regular physical activity is a cornerstone of cardiovascular health and longevity, the spectrum of cardiovascular responses to exercise extends beyond merely beneficial adaptation. The increasing recognition of exercise-induced arrhythmias, maladaptive remodeling, and sudden cardiac death, particularly among athletes, has sharpened the need for nuanced, mechanistically informed research. This research topic, *Cardiovascular Responses to Exercise: Clinical and Pathological Perspectives in Athletes*, was designed to address this complexity by bringing together clinical, translational, and mechanistic studies that examine how exercise influences cardiovascular structure, function, and risk.

The eight articles published in this special topic collectively highlight the dual nature of exercise as both a potent therapeutic intervention and a contributor to pathological cardiovascular remodeling under specific conditions. Importantly, the articles published in this special issue highlight the value of advanced imaging, rigorous physiological assessment, and individualized exercise prescriptions to distinguish adaptive from maladaptive responses.

## Exercise-induced cardiac remodeling: adaptive vs. maladaptive

Several contributions focused on structural and functional cardiac remodeling in response to sustained exercise. The study of amateur marathon runners by Wang et al. provided critical insight into left ventricular hypertrophy (LVH) as a potential marker of excessive cardiac remodeling. Using pressure–strain loop–derived myocardial work indices, the authors demonstrated that moderate exercise adaptations are associated with higher global myocardial work. However, excessive weekly running distance correlates with reduced myocardial work efficiency and increased wasted work, particularly in male athletes. These findings refine the concept of the “athlete's heart” by suggesting that myocardial efficiency may serve as a more sensitive indicator of maladaptive remodeling compared with myocardial size and mass alone.

Complementing this work, Christou et al. presented a pediatric case report on a juvenile athlete with a high number of premature ventricular contractions, highlighting the clinical consequences of exercise-associated arrhythmias and the importance of understanding individualized management strategies. The successful resolution of arrhythmia-induced cardiomyopathy in later juvenile years through the use of low-dose flecainide in conjunction with beta-blocker therapy suggests that pharmacological strategies, when carefully selected, can safely preserve athletic participation in young individuals without resorting to invasive interventions.

## Exercise as a personalized therapy in cardiovascular disease

A large portion of this research topic addresses exercise as a therapeutic modality in cardiovascular disease, particularly following myocardial infarction (MI). Two large-scale meta-analyses [by Zhang et al. and Bo Yu et al.] reinforced the beneficial role of structured exercise training in improving cardiac function post-MI. Exercise was shown to significantly enhance left ventricular ejection fraction and functional capacity, with resistance exercise demonstrating particularly strong effects on cardiac remodeling outcomes. These findings challenge the traditional dominance of aerobic exercise in cardiac rehabilitation programs and support a more diversified and nuanced approach.

At the mechanistic level, a preclinical study by Zhang et al. employing longitudinal cardiac magnetic resonance imaging and 18F-FDG PET provided compelling evidence in a rat model of MI that exercise, when started early after MI, improves myocardial function by enhancing glucose metabolism, reducing fibrosis, and attenuating inflammation in the ischemic border zone. The observed upregulation of the glucose transporter GLUT4 and the glycolytic enzyme PFKFB3 links functional recovery to metabolic reprogramming, offering a mechanistic framework that bridges clinical imaging phenotypes with molecular adaptations.

Finally, a systematic review and meta-analysis of “exercise snacks”, short, high-intensity bouts of physical activity performed intermittently throughout the day, introduced an emerging paradigm for improving cardiometabolic health in individuals with time constraints Chen et al. This work broadens the applicability of exercise-based interventions beyond traditional training programs, with potential relevance for both prevention and cardiac rehabilitation.

## Cardiorespiratory fitness, body composition, and systemic disease

Beyond cardiac structure and function, several articles emphasize cardiorespiratory fitness (CRF) as a unifying determinant of cardiovascular risk across populations. An observational study [by Popp and Jesch] in healthy adults utilized dual-energy x-ray absorptiometry and VO2max estimates to demonstrate that excess adiposity (fat mass adjusted for height) is negatively associated with CRF, independent of total energy expenditure. These findings reinforce the importance of body composition, rather than merely body mass index, in interpreting cardiovascular fitness and risk.

Extending this concept to a clinical population, Wang et al. examined a large cohort of cancer patients and revealed a robust, inverse association between CRF and adverse cardiovascular outcomes, including heart failure, atrial fibrillation, and cancer therapy–related cardiotoxicity. These findings further underscore the importance of targeted exercise interventions in vulnerable populations.

## Future directions and clinical implications

Taken together, the studies in this research topic emphasize that cardiovascular responses to exercise are highly context-dependent and are shaped by exercise dose, modality, individual susceptibility, and underlying disease. Advanced imaging and physiological metrics are increasingly critical for identifying early markers of maladaptation, and mechanistic studies continue to illuminate the molecular pathways through which exercise exerts its effects.

Future research should focus on integrating imaging, biomarkers, mechanisms, and functional assessments to refine cardiovascular risk stratification in athletes and clinical populations alike. The field would benefit greatly from additional animal exercise models examining the mechanisms of the cardiovascular effects of exercise. Of equal importance is the development of personalized exercise prescriptions that maximize therapeutic benefit while minimizing the pathological risk.

The editors of this research topic hope that this serves as a valuable resource for clinicians, scientists, and sports medicine professionals seeking to better understand the complex interplay between exercise and cardiovascular health; ultimately allowing for the translation of this knowledge into safer and more effective exercise strategies throughout one's lifespan.

